# Associations of Long-Term Exposure to Temperature Variability with Glucose Metabolism: Results from KORA F4 and FF4

**DOI:** 10.1021/acs.est.5c04956

**Published:** 2025-11-07

**Authors:** Wenli Ni, Siqi Zhang, Christian Herder, Susanne Breitner-Busch, Kathrin Wolf, Minqi Liao, Nikolaos Nikolaou, Regina Pickford, Wolfgang Koenig, Wolfgang Rathmann, Lars Schwettmann, Michael Roden, Barbara Thorand, Annette Peters, Alexandra Schneider

**Affiliations:** 1 Institute of Epidemiology, Helmholtz Zentrum München−German Research Center for Environmental Health, Neuherberg D-85764, Germany; 2 Institute for Medical Information Processing, Biometry, and Epidemiology, Pettenkofer School of Public Health, LMU Munich, Munich 81377, Germany; 3 Institute for Clinical Diabetology, German Diabetes Center, Leibniz Center for Diabetes Research at Heinrich Heine University Düsseldorf, Düsseldorf 40225, Germany; 4 Division of Endocrinology and Diabetology, Medical Faculty and University Hospital Düsseldorf, Heinrich Heine University Düsseldorf, Düsseldorf 40204, Germany; 5 German Center for Diabetes Research (DZD e.V.), München-Neuherberg, Munich D-85764, Germany; 6 School of Medicine and Health, German Heart Centre, TUM University Hospital, Technical University of Munich, Munich 80636, Germany; 7 Munich Heart Alliance, German Centre for Cardiovascular Research (DZHK e.V.), Partner Site Munich, Munich 80802, Germany; 8 Institute of Epidemiology and Medical Biometry, University of Ulm, Ulm 89081, Germany; 9 Institute for Biometrics and Epidemiology, German Diabetes Centre, Leibniz Center for Diabetes Research at Heinrich Heine University Düsseldorf, Düsseldorf 40225, Germany; 10 Institute of Health Economics and Health Care Management, Helmholtz Zentrum München−German Research Centre for Environmental Health, Neuherberg 85764, Germany; 11 Department of Health Services Research, School of Medicine and Health Sciences, Carl von Ossietzky Universität Oldenburg, Oldenburg 26129, Germany

**Keywords:** temperature variability, glucose metabolism, fasting insulin, HOMA-IR, HbA1c, QUICKI, long-term effects

## Abstract

The impact of rising temperature variability driven by climate change on metabolic health remains understudied, especially considering the global increase in diabetes prevalence, with long-term effects on glucose metabolism unexplored. This study investigated associations between long-term temperature variability exposure and glucose metabolism in a population-based cohort of 2997 participants (4954 observations) over a 7-year period from KORA F4 and FF4 cohorts in Augsburg, Germany. Long-term exposure to temperature variability was estimated as the standard deviation of the daily mean air temperature over the 365-day period preceding each examination. We applied generalized estimating equations to examine the longitudinal associations between long-term exposure to temperature variability and multiple glucose metabolism biomarkers: fasting glucose, 2h glucose, fasting insulin, homeostasis model assessment of insulin resistance (HOMA-IR), homeostasis model assessment of β-cell function (HOMA-B), quantitative insulin sensitivity check index (QUICKI), and glycated hemoglobin (HbA1c). We found that a 1 °C higher temperature variability was significantly associated with higher fasting insulin, HOMA-IR, and HbA1c with % changes (95% CI) of 2.62 (0.79; 4.49), 2.81 (0.79; 4.87), and 2.38 (1.97; 2.79), respectively, and lower QUICKI (−0.41 [−0.70; −0.11]). These findings suggest that increasing temperature variability exposure may contribute to metabolic dysfunction, potentially accelerating the global diabetes epidemic.

## Graphical abstract

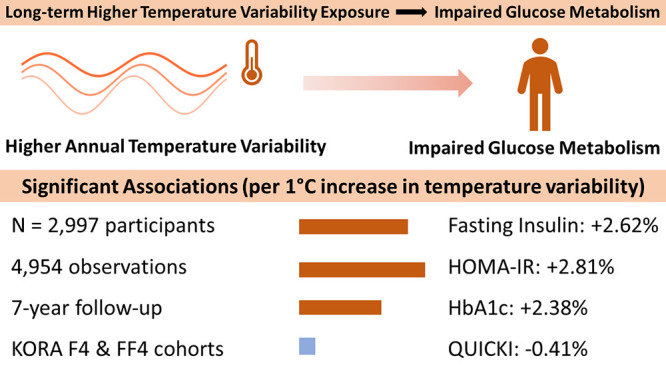

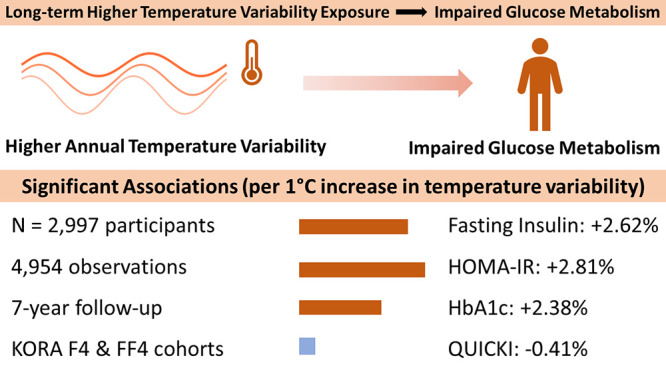

## 1 Introduction

Climate change is an increasingly pressing concern for nations across the globe. Beyond raising global average temperatures, climate change also leads to greater temperature variability, resulting in amplified fluctuations across both seasonal and interannual timescales.^1–5^ Climate model projections indicate that temperature variability increases by approximately 15% per degree of global warming in regions such as Amazonia and Southern Africa and by about 10% in subtropical hotspots of the Northern Hemisphere, mainly due to mechanisms such as soil drying and shifts in atmospheric structure.^1^ A review study further revealed that surface air temperature variability on longer timescales, such as annual variability, appears to be increasing.^3^ Despite these trends, limited research has examined the long-term health impacts of increased temperature variability.^3^ Emerging epidemiological evidence suggests that long-term exposure to greater temperature variability is associated with increased mortality in older adults^6^ and a higher prevalence of chronic conditions such as cardiovascular disease, respiratory illnesses, arthritis, and cataracts.^7^ These findings highlight the possibility that temperature variability may constitute a significant health risk.

Diabetes is the eighth leading cause of disability-adjusted life-years globally and continues to increase in prevalence.^8,9^ Biomarkers of glucose metabolism including fasting glucose, 2 h glucose (2h glucose) in an oral glucose tolerance test, fasting insulin, homeostasis model assessment of insulin resistance (HOMA-IR), homeostasis model assessment of β-cell function (HOMA-B), quantitative insulin sensitivity check index (QUICKI), and glycated hemoglobin (HbA1c) are of paramount importance in the evaluation and comprehension of the progression of diabetes.^10^ Previous studies suggest that low or high temperature exposure may have a detrimental effect on glucose metabolism and diabetes-related mortality.^11–14^ Yet, although temperature variability itself is growing under climate change, its long-term impact on glucose metabolism biomarkers has not been investigated. Given the long-term progression of diabetes and related metabolic disorders, examining the chronic effects of temperature variability over extended periods can provide important insights into chronic physiological adaptations and cumulative health impacts.

Therefore, we aimed to assess the associations between long-term exposure to temperature variability and glucose metabolism biomarkers with fasting glucose, 2h glucose, fasting insulin, HOMA-IR, HOMA-B, QUICKI, and HbA1c repeatedly measured seven years apart.

## 2 Methods

### 2.1 Study Design and Participants

Data for this longitudinal analysis were obtained from the population-based KORA (Cooperative Health Research in the Region of Augsburg) studies F4 (2006–2008) and FF4 (2013–2014), both follow-up examinations of the fourth survey of the population-based KORA study (KORA S4, 1999–2001) conducted in the city of Augsburg, Germany, and its two surrounding districts.^15^ The framework, design, measurement methods, and data collection of the KORA cohort have been described elsewhere.^15–17^ The present study included participant observations with available data on fasting glucose, 2h glucose, fasting insulin, HOMA-IR, HOMA-B, QUICKI, or HbA1c measurements if participants were not taking glucose-lowering medication and if their blood sample was drawn before 11:00 am. For the analysis of 2h glucose biomarkers, participants with clinically diagnosed diabetes were excluded, as they did not undergo the oral glucose tolerance test (OGTT), which is required to obtain 2h glucose measurements.

The present study included 4954 observations of 2997 participants, comprising of 2880 who took part in KORA F4 and 2074 who took part in KORA FF4. Out of these, 1957 participants (65.3%) completed both examinations, while 1040 participants (34.7%) attended one examination. Thus, our sample consists of participants who joined at F4 only, participants who newly joined at FF4, and participants who participated in both waves.

The study complied with the Declaration of Helsinki and was approved by the Ethics Committee at the Bavarian Chamber of Physicians (Munich, Germany). All participants gave their written informed consent.

### 2.2 Exposure Assessment

Assessment of air temperature has been described in detail previously.^18^ In brief, spatiotemporal regression-based models were used to simulate the countrywide high-resolution (1 × 1 km) daily air temperature data, consisting of the mean, minimum, and maximum temperatures. Three-stage models were employed to generate historical air temperature data that offer a broad temporal and spatial coverage. In the initial step, a linear mixed model was formulated that incorporated daily random intercepts and slopes for land surface temperature (LST) and adjusted with spatial predictors to estimate air temperature in grid cells that contained both air temperature measurements and LST data. In the subsequent stage, this model was employed to estimate air temperature for grid cells, which had available LST data but no air temperature measurements. The third step consisted of regressing the second stage predictions against interpolated air temperature values to acquire air temperature all across the country. The models’ estimations when evaluated through a 10-fold cross-validation against ground measurements in the stations’ locations around Germany showed high precision (*R* ^2^ ranging from 0.91 to 0.98) and low errors (root-mean-square error [RMSE] from 1.03 to 2.02 °C). In addition, an extensive validation was conducted specifically for Augsburg, where our participants live, against a dense (around 80 HOBO-Logger sensors) and independent monitoring network, further supporting the reliability of the temperature estimates in the KORA study region (0.95 ≤ *R* ^2^ ≤ 0.99 and 1.07 °C ≤ RMSE ≤ 1.80 °C).

Daily temperature data were assigned to each participant based on their residential address at the day of examination (blood draw). Residential addresses were geocoded and matched to the nearest 1 × 1 km grid cell from our high-resolution temperature data set. Residential address information for each participant in the initial KORA S4 survey was obtained from official local registration office records. For follow-up examinations (KORA F4 and KORA FF4), addresses were updated if invitation letters were undeliverable, through active contact with participants (by phone) or new data from registration offices. Therefore, we obtained geocoded addresses that were valid for each participant at the time of their clinical examination. In addition, we had indicators for relocation between survey waves and variables estimating residence duration at each follow-up phase. Temperature data coverage was 100% complete over the study period, ensuring no gaps in exposure assessment. For each participant, long-term temperature variability exposure was calculated as the standard deviation (SD) of daily mean temperatures over the 365-day period immediately preceding their examination date. This individual-specific temporal exposure window ensures that each participant’s temperature variability exposure reflects their unique examination timing rather than a fixed calendar period.

Through the application of land-use regression (LUR) models, the average mean concentrations of ozone (O_3_), particulate matter with an aerodynamic diameter of ≤2.5 μm (PM_2.5_), and nitrogen dioxide (NO_2_) were determined.^19^ Between March 6, 2014 and April 7, 2015, three 2-week measurements were accomplished at 20 locations within the KORA study area throughout the warm, cold, and intermediate seasons, to obtain annual average air pollutant concentrations. Subsequently, regression of the obtained annual average concentrations in 2014–2015 against geographic information system-based spatial predictors was then used to construct LUR models, which were further applied to the residential addresses of participants to assess their exposure levels.

### 2.3 Measurement of Biomarkers of Glucose Metabolism

Prior to the visit to the KORA study center, participants were requested to fast for at least 8 h and to not consume anything except mineral water. Furthermore, physical exertion and smoking were prohibited on the day before and on the morning of the sample collection. The blood samples were acquired after a rest of 5 min with the participants in a sitting position.

We assessed fasting glucose, fasting insulin, 2h glucose, HOMA-IR, HOMA-B, QUICKI, and HbA1c. Fasting glucose was defined as the concentration of glucose in blood after fasting for at least 8 h, reflecting baseline glycemic status. Fasting insulin, measured in circulatory blood after fasting for at least 8 h, was used as an indicator of basal insulin secretion. The 2h glucose measurement refers to the plasma glucose concentration determined 2 h after a standard OGTT, thereby reflecting postchallenge glycemic response.^20^ HOMA-IR is an index calculated from fasting glucose and fasting insulin concentrations to estimate insulin resistance.^10,20^ HOMA-B is derived from fasting glucose and fasting insulin to provide an estimate of pancreatic β-cell function.^10^ QUICKI is a logarithmic index calculated from fasting glucose and insulin, used to estimate insulin sensitivity.^10,20,21^ Finally, HbA1c reflects average blood glucose levels over the previous two to three months and serves as an indicator of chronic glycemic control.^22,23^ Detailed descriptions of the methods used to measure biomarkers of glucose metabolism, including measurements of fasting glucose, fasting insulin, 2h glucose, HOMA-IR, HOMA-B, QUICKI, and HbA1c, are provided in Table S1 and have also been previously described.^24–27^ The assessment of covariates is provided in Text S1.

### 2.4 Statistical Analysis

We performed descriptive analyses to summarize the characteristics of the study population, as well as the distributions of exposure and glucose metabolism variables. Continuous variables were summarized using means and SD or medians and interquartile ranges (IQR). Categorical variables were presented as frequencies and percentages. Spearman correlation coefficients were calculated separately to assess correlations among exposure variables and among glucose metabolism variables.

We applied generalized estimating equations (GEE) to explore the longitudinal associations between long-term exposure to temperature variability at the participants’ home address and repeatedly assessed biomarkers of glucose metabolism: fasting glucose, 2h glucose, fasting insulin, HOMA-IR, HOMA-B, QUICKI, and HbA1c. To exploit within-person longitudinal information provided by repeated measures and to maximize statistical power by including nonrepeated measures, our main analysis includes all available observations from KORA F4 and FF4. Natural log-transformation of biomarkers of glucose metabolism was conducted for the purpose of improving normality of residuals. A preliminary analysis was carried out to explore the exposure–response functions of temperature variability and biomarkers of glucose metabolism by adding the temperature variability as a spline with four degrees of freedom and using the likelihood ratio test (LR test) to test for nonlinearity. We found no remarkable deviations from linearity with regard to the temperature variability on fasting glucose, 2h glucose, fasting insulin, HOMA-IR, HOMA-B, and QUICKI (Table S2). Furthermore, the exposure–response function for HbA1c suggested a monotonic association, with the LR test indicating some deviations from linearity and a particularly increased risk at temperature variability values above approximately 7.5 °C (Figure S1). To be consistent across biomarkers, temperature variability was ultimately incorporated linearly into the GEE models. Additionally, given the threshold at approximately 7.5 °C observed in the exposure–response curve for HbA1c, we also conducted a segmented regression analysis with a knot at 7.5 °C as a secondary analysis. Based on prior literature and our own experience,^24^ we adjusted the models for age, sex, body mass index [BMI], education, cigarette smoking, alcohol consumption, physical activity, occupational status, time of blood withdrawal (hours), season of blood withdrawal (spring, summer, fall, and winter), year of blood withdrawal, and high-sensitivity C-reactive protein (hsCRP) levels, which have been previously correlated with insulin resistance.^28^

Effect modification analyses were performed by including an interaction term between temperature variability and the following potential effect modifiers: age (<65 vs ≥65 years), sex (male vs female), (pre)­diabetes status (normal glucose tolerance vs prediabetes/diabetes), physical activity (low vs medium/high), overweight/obesity (BMI <25 kg/m^2^ vs ≥25 kg/m^2^), and smoking status (current vs former/never smoker).

We performed multiple sensitivity analyses to evaluate the robustness of the results. First, we included only those participant observations that had complete data for all biomarkers. Second, we further adjusted for annual average temperature in the main model. Third, to account for the confounding from short-term effects, we controlled for short-term exposure to temperature variability, defined as the SD of the temperature over a 2-day period (lagged by 0–1 days), and the average temperature, calculated as the moving average of the temperature over the same 2-day period (also lagged by 0–1 days), in the model. Fourth, we further adjusted for total cholesterol, high-density lipoprotein cholesterol, and waist–hip ratio. Fifth, we used the SD of the 365-day moving average of daily minimum air temperature (Tmin) and the SD of the 365-day moving average of daily maximum air temperature (Tmax) before the blood draw as substitutes for the variability of mean temperature. Sixth, we excluded participants who moved during the study period to minimize the potential for exposure misclassification. Seventh, to avoid overestimation of the association due to extreme values, we excluded biomarkers of glucose metabolism that fell below the first quartile of the data minus 1.5 times the IQR or above the third quartile of the data plus 1.5 times the IQR. Eighth, we included only participants who had repeated measurements of biomarkers of glucose metabolism (*N* = 1957 participants) in the analysis. Ninth, to account for potential confounding by air pollutants, we separately adjusted for the annual average concentrations of PM_2.5_, NO_2_, and O_3_. To further control for confounding by short-term air pollution exposure, we also conducted separate adjustments for the moving average (lag 0–1 days) of PM_2.5_, NO_2_, and O_3_. We also limited the analysis to unemployed participants, who are more likely to spend most of their time at home, to evaluate the robustness of our findings. Finally, to determine whether the associations held when using clinically meaningful categories and to further contextualize the clinical significance of our observed effect sizes, we conducted an additional sensitivity analysis by classifying participants into binary categories (normal vs prediabetic/diabetic) for fasting glucose (≥100 mg/dL), 2h glucose (≥140 mg/dL), and HbA1c (≥39 mmol/mol) using American Diabetes Association (ADA)-recommended thresholds.^29^ For other markers lacking universally accepted cutoffs, we applied literature-based thresholds for insulin resistance or metabolic syndrome: HOMA-IR > 2,^30,31^ fasting insulin > 12.2 μIU/mL,^31,32^ HOMA-B < 94.74,^33^ and QUICKI < 0.33.^31,34^

Effect estimates are displayed as a percent change of the geometric mean with 95% confidence intervals (CIs) for each 1 °C rise in temperature variability. For sensitivity analyses that used glucose metabolism outcomes categorized as binary variables, effect estimates are reported as odd ratios (ORs) with 95% CIs. Multiple testing was corrected using Benjamini–Hochberg false discovery rate (FDR) methods with a significance level of *p* < 0.05. All statistical analyses were performed using R software version 4.1.2.

## 3 Results

### 3.1 Study Population, Glucose Metabolism Biomarkers, and Exposure Data

The descriptive characteristics for the study population at each examination point are shown in Table 1. For KORA F4 and KORA FF4, the mean ages were 55.4 and 59.5 years, respectively, and 47.7 and 47.5% of the participants were male, respectively.

**Table 1:** Descriptive Statistics of Participant Characteristics at Each Examination^a^

	mean ± SD/median [IQR]/*N* (%)	
	KORA F4, 2006–2008 (*N* = 2880)	KORA FF4, 2013–2014 (*N* = 2074)
Age (years)	55.4 ± 13.1	59.5 ± 12.2
Sex (male)	1374 (47.7%)	986 (47.5%)
BMI (kg/m^2^)	27.4 ± 4.7	27.5 ± 4.9
missing	11 (0.4%)	2 (0.1%)
Education (years)	11.8 ± 2.7	12.0 ± 2.7
missing	5 (0.2%)	5 (0.2%)
Occupation (employed)	1655 (57.5%)	1213 (58.5%)
missing	1 (0.0%)	2 (0.1%)
Smoking status		
never	1205 (41.8%)	877 (42.3%)
former smoker	1149 (39.9%)	867 (41.8%)
current smoker	522 (18.1%)	330 (15.9%)
missing	4 (0.1%)	0 (0%)
Physical activity		
low	894 (31.0%)	553 (26.7%)
nedium (∼1 h per week)	1265 (43.9%)	960 (46.3%)
high (∼2 h per week)	718 (24.9%)	561 (27.0%)
missing	3 (0.1%)	0 (0%)
Alcohol consumption (g/day)*	5.71 [20.0]	5.71 [21.6]
missing	3 (0.1%)	1 (0.0%)
hsCRP (mg/L)*	1.16 [2.01]	1.17 [1.97]
missing	16 (0.6%)	14 (0.7%)
Waist–hip ratio	0.88 ± 0.09	0.90 ± 0.09
missing	9 (0.3%)	3 (0.1%)
Total cholesterol (mg/dL)	217 ± 39.3	218 ± 39.2
missing	0 (0%)	2 (0.1%)
High-density lipoprotein cholesterol (mg/dL)	56.3 ± 14.5	66.3 ± 18.8
missing	1 (0.0%)	2 (0.1%)
(Pre)diabetes status		
normal glucose tolerance	1785 (62.0%)	1083 (52.2%)
prediabetes/type 2 diabetes	1018 (35.3%)	913 (44.0%)
missing	77 (2.7%)	78 (3.8%)

a Note: Missing data were addressed using complete case analysis. *Median [IQR].

The median levels of glucose metabolism biomarkers in KORA F4 were as follows: fasting glucose, 93.0 mg/dL; 2h glucose, 104.0 mg/dL; fasting insulin, 8.7 μIU/mL; HOMA-IR, 2.0; HOMA-B, 104.5; QUICKI, 0.344; and HbA1c, 36.0 mmol/mol (Table 2). Corresponding median values in KORA FF4 were as follows: fasting glucose, 97.0 mg/dL; 2h glucose, 104.0 mg/dL; fasting insulin, 8.9 μIU/mL; HOMA-IR, 2.1; HOMA-B, 95.1; QUICKI, 0.340; and HbA1c, 35.0 mmol/mol (Table 2). Temporal trends in glucose metabolism biomarkers are shown in Figures S2 and S3, demonstrating that the levels of these biomarkers exhibited no marked seasonal or cyclical patterns during the study period. Figure S4 shows that there were weak to moderate correlations between these glucose metabolism biomarkers, except for strong correlations between fasting insulin and HOMA-IR, HOMA-B, and QUICKI, and between HOMA-IR and QUICKI.

**Table 2:** Glucose Metabolism Biomarkers at Each Examination^a^

	KORA F4, 2006–2008 (*N* = 2880)		KORA FF4, 2013–2014 (*N* = 2074)	
	median [IQR]	mean ± SD	median [IQR]	mean ± SD
fasting glucose (mg/dL)	93.0 [13.0]	95.8 ± 14.4	97.0 [14.0]	98.8 ± 13.9
2h glucose (mg/dL)	104.0 [41.0]	112 ± 39.1	104.0 [42.0]	113.0 ± 40.9
fasting insulin (μIU/mL)	8.7 [6.3]	10.6 ± 7.1	8.9 [6.9]	10.6 ± 6.8
HOMA-IR	2.0 [1.7]	2.6 ± 2.1	2.1 [1.9]	2.7 ± 2.1
HOMA-B	104.5 [67.4]	121 ± 70.8	95.1 [65.3]	109 ± 61.5
QUICKI	0.344 [0.041]	0.344 ± 0.031	0.340 [0.042]	0.342 ± 0.032
HbA1c (mmol/mol)	36.0 [6.0]	36.3 ± 5.1	35.0 [5.0]	35.9 ± 4.8

a Note: HOMA-IR, homeostasis model assessment of insulin resistance; HOMA-B, homeostasis model assessment of β-cell function; QUICKI, quantitative insulin sensitivity check index; HbA1c, glycated hemoglobin. Fasting glucose missing data: KORA F4, 23 (0.8%); KORA FF4, 17 (0.8%). 2h glucose missing data: KORA F4, 133 (4.6%); KORA FF4, 128 (6.2%). Fasting insulin missing data: KORA F4, 53 (1.8%); KORA FF4, 17 (0.8%). HOMA-IR missing data: KORA F4, 55 (1.9%); KORA FF4, 18 (0.9%). HOMA-B missing data: KORA F4, 55 (1.9%); KORA FF4, 18 (0.9%). QUICKI missing data: KORA F4, 55 (1.9%); KORA FF4, 18 (0.9%). HbA1c missing data: KORA FF4, 6 (0.3%).

For the KORA F4 study conducted between 2006 and 2008, the mean temperature variability experienced by participants (calculated as the SD of daily mean temperatures over the 365 days preceding each participant’s examination date) was 6.9 ± 0.7 °C (mean ± SD, Table 3). In the KORA FF4 study from 2013 to 2014, the corresponding value was 7.3 ± 0.6 °C (mean ± SD, Table 3). Table S3 presents the annual average temperature and annual temperature variability for the Augsburg region for each calendar year of the study period.

**Table 3:** Descriptive Statistics of Air Temperatures and Air Pollutants during the KORA F4 and FF4 Study Period

	KORA F4, 2006–2008							KORA FF4, 2013–2014						
	mean	SD	5%	25%	median	75%	95%	mean	SD	5%	25%	median	75%	95%
temperature variability (°C)	6.9	0.7	6.0	6.4	6.7	7.1	8.6	7.3	0.6	6.4	6.7	7.6	7.8	8
annual average temperature (°C)	9.7	0.8	8.3	9.1	9.7	10.3	11	8.9	0.6	8	8.4	8.8	9.4	9.9
PM_2.5_ (μg/m^3^)	39.1	2.3	35.3	37.4	39.2	40.8	42.7	39.1	2.3	35.3	37.5	39.2	40.9	42.7
NO_2_ (μg/m^3^)	11.7	1	9.9	11	11.8	12.4	13.1	11.6	1	9.8	11	11.8	12.4	13.1
O_3_ (μg/m^3^)	14.2	4.4	7.3	10.6	13.8	17.5	21.9	13.9	4.3	7.3	10.4	13.5	17.1	21.4

Figure S5 shows generally weak correlations between air temperature and pollutant variables (*r*: −0.26 to 0.27), except for temperature variability and annual average temperature (*r* = −0.43 in KORA F4 and *r* = −0.64 in KORA FF4) and PM_2.5_ and NO_2_ (*r* = 0.79 in KORA F4 and *r* = 0.80 in KORA FF4).

### 3.2 Associations of Long-Term Exposure to Temperature Variability with Glucose Metabolism

The associations of long-term exposure to temperature variability with fasting glucose, 2h glucose, fasting insulin, HOMA-IR, HOMA-B, QUICKI, and HbA1c are shown in Figure 1. We found that a 1 °C higher temperature variability was significantly associated with higher fasting insulin, HOMA-IR, and HbA1c (% changes [95% CI]: 2.62 [0.79; 4.49], 2.81 [0.79; 4.87], and 2.38 [1.97; 2.79], respectively) and lower QUICKI (−0.41 [−0.70; −0.11]), after adjustment for multiple testing. However, we did not find statistically significant associations between temperature variability and fasting glucose, 2h glucose, and HOMA-B, after adjustment for multiple testing (*p*-adjusted >0.05).

**Figure 1:**
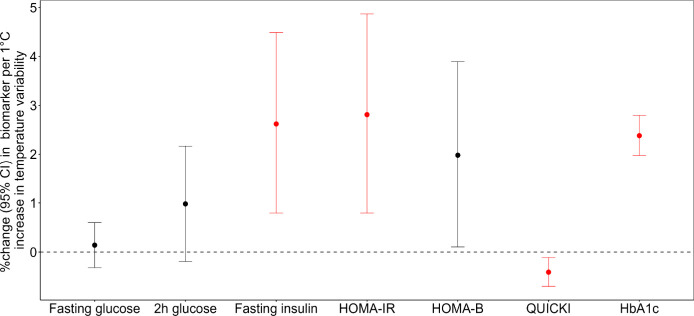
Estimation of percent changes [95% CI] in geometric means of glucose metabolism biomarkers with a 1 °C increase in temperature variability. Note: Error bars in red demonstrate significant associations after multiple testing (adjusted *p*-value <0.05).

In the segmented regression analysis for HbA1c with a knot at 7.5 °C (Table S4), we found that for temperature variability values at or above 7.5 °C, each 1 °C increase was associated with a significant increase in HbA1c (% change [95% CI]: 6.71 [5.78, 7.65]), whereas no significant association was observed for values below 7.5 °C.

### 3.3 Effect Modification

Figure 2 and Figure S6 show the effect modification of long-term exposure to temperature variability on glucose metabolism. For long-term temperature exposure, we found trends toward stronger effects on glucose metabolism among individuals aged 65 years and older, compared to those under 65 years, although these differences were not statistically significant. Moreover, there were no significant effect modifications by sex, (pre)­diabetes status, physical activity, overweight and obesity, or smoking status.

**Figure 2:**
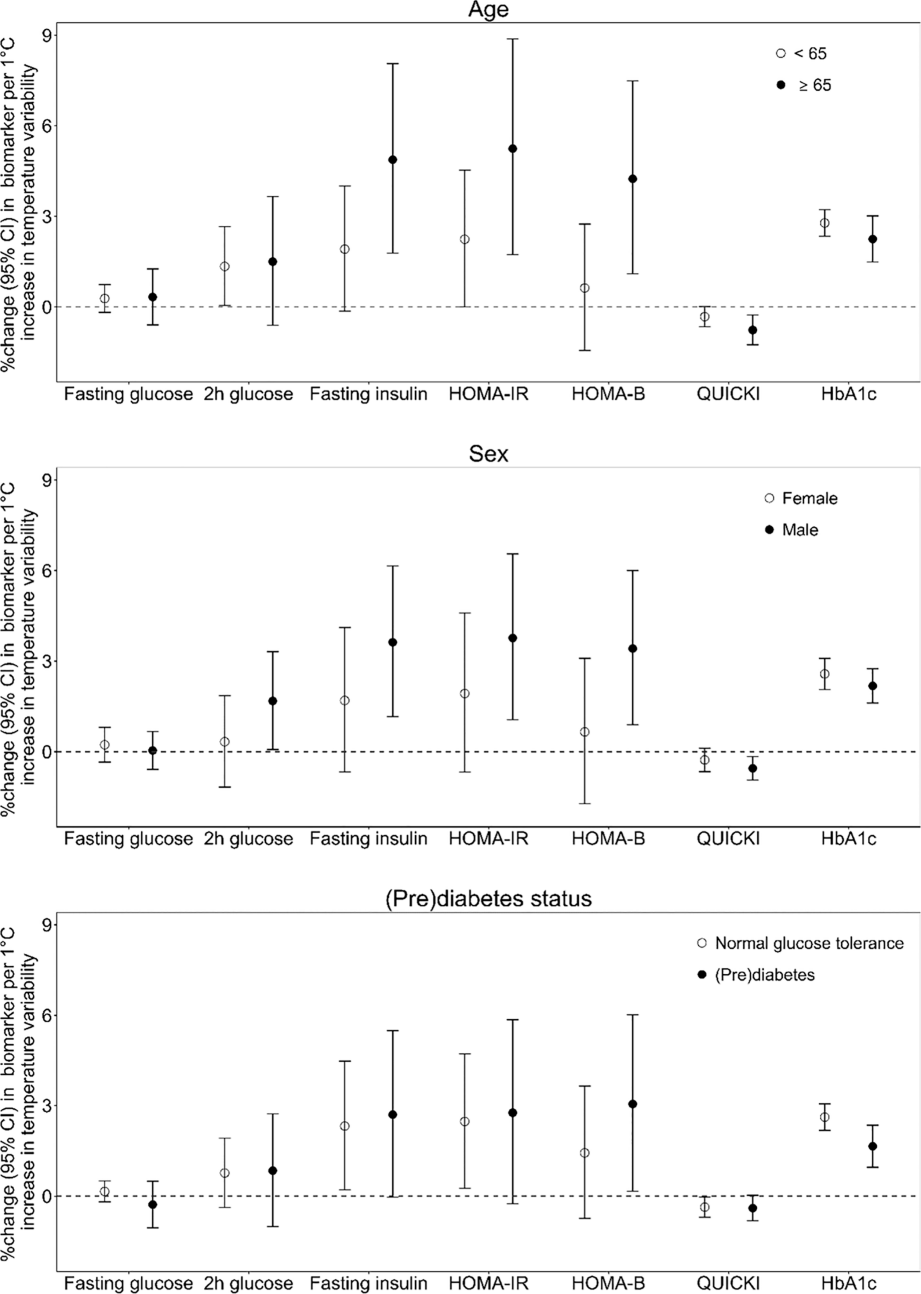
Estimation of percent changes in geometric means of glucose metabolism biomarkers with a 1 °C increase in temperature variability modified by age, sex, and (pre)­diabetes status.

### 3.4 Sensitivity Analysis

Generally, the longitudinal associations between long-term exposure to temperature variability and glucose metabolism remained consistent across various sensitivity analyses (Figure S7). We observed analogous associations when we included only participant observations with complete data for all analyzed outcomes. Similar results were also noted when the annual average temperature was additionally factored into the model; although the effect estimate of temperature variability on HbA1c decreased, the association remained statistically significant. Comparable results were observed when short-term temperature variability and average temperature were additionally considered in the model. The associations remained consistent even when additional adjustments were made for total cholesterol, high-density lipoprotein cholesterol, and waist–hip ratio. Moreover, the effect estimates were consistent when additional adjustments were made for both annual and short-term air pollutants exposures (PM_2.5_, NO_2_, and O_3_). Furthermore, similar effect estimates were obtained when the SD of minimum or maximum temperatures were used. The results remained unaffected by the exclusion of participants who relocated during the study period, the exclusion of outliers, the limitation to participants with repeated measurements of glucose metabolism, and by restricting the analysis to unemployed participants. Finally, sensitivity analysis using binary clinical or literature-based categories (Figure S8) revealed that higher long-term temperature variability exposure was significantly associated with increased odds of abnormal fasting insulin (>12.2 μIU/mL; OR = 1.23, 95% CI: 1.11–1.37), HOMA-IR (>2; OR = 1.15, 95% CI: 1.04–1.27), QUICKI (<0.33; OR = 1.13, 95% CI: 1.01–1.25), and HbA1c (≥39 mmol/mol; OR = 1.45, 95% CI: 1.30–1.62) per 1 °C increase in temperature variability. No significant association was observed for fasting glucose, 2h glucose, and HOMA-B. These findings were consistent with those from the main analysis, supporting the robustness and clinical relevance of our results.

## 4 Discussion

Our study demonstrated that long-term exposure to increased temperature variability was significantly associated with higher levels of fasting insulin, HOMA-IR, and HbA1c and lower levels of QUICKI. To the best of our knowledge, this is the first investigation to assess the associations between long-term exposure to temperature variability and glucose metabolism over an extended period, providing novel insights into potential mechanisms through which temperature variability, as a potential consequence of climate change, may influence metabolic health.

Fasting insulin, HOMA-IR, QUICKI, and HbA1c have a substantial role in the progression of type 2 diabetes and are likewise correlated with an increased risk of cardiovascular disease.^35,36^ Our study found that a 1 °C increase in temperature variability was significantly associated with an increase of 2.62% in fasting insulin, 2.81% in HOMA-IR, and 2.38% in HbA1c, as well as a decrease of 0.41% in QUICKI. These associations were also consistently observed using clinically relevant thresholds, with each 1 °C increase in temperature variability associated with 15% higher odds of HOMA-IR > 2, 23% higher odds of fasting insulin > 12.2 μIU/mL, 45% higher odds of HbA1c ≥ 39 mmol/mol, and 13% higher odds of QUICKI < 0.33. For example, in our study population (KORA F4 and FF4), the prevalence of abnormal HbA1c (≥39 mmol/mol) was 24.2%. A 1 °C rise in temperature variability corresponds to an absolute increase in the prevalence of abnormal HbA1c from 24.2% to approximately 31.6%, assuming the association is causal. Furthermore, historical climate data indicate a gradual increase in temperature variability in Germany over recent decades;^4^ a similar trend was observed in our study region, where the average temperature variability exposure (calculated as the SD of daily mean temperatures over the 365 days preceding each participant’s examination) was 6.9 °C for participants examined in 2006–2008 and 7.3 °C for those in 2013–2014. Although these individual-level effect sizes are modest, their application at the entire population level, especially in the context of demographic aging and ongoing climate change, could translate to a meaningful increase in metabolic disease burden. These results suggest that higher temperature variability may contribute to increased insulin resistance and a higher risk of type 2 diabetes and related metabolic diseases. While more research is needed, especially in diverse populations and climates, it may be warranted for future public health strategies to consider environmental factors such as temperature variability in the broader context of metabolic disease prevention and management.

We did not observe significant associations between temperature variability and fasting glucose, 2h glucose, or HOMA-B. The underlying reasons for this pattern are not fully understood. It is possible that temperature variability may more strongly affect specific pathways of glucose metabolism, such as those related to insulin resistance (as reflected by fasting insulin, HOMA-IR,^10,20^ and QUICKI^10,20,21^ ) or long-term glucose regulation (HbA1c^22,23^ ), rather than short-term glucose concentrations (fasting glucose and 2h glucose) or pancreatic β-cell function (HOMA-B^10^). Further research is needed to clarify these differential effects and to elucidate the mechanisms involved.

It is important to note that higher fasting insulin, higher HOMA-IR, and lower QUICKI mainly reflect hepatic insulin resistance whereas the assessment of whole-body insulin sensitivity would require additional data from a 5-point OGTT or other tests. Despite this limitation, our research contributes to a better understanding of the underlying mechanisms linking climate change to the ongoing rise in cardiometabolic diseases worldwide.^9,37^ In the context of climate change, characterized by increased temperature variability and extreme weather events,^1,2^ our findings underscore the importance of developing comprehensive strategies that simultaneously address climate change mitigation and public health protection, particularly in relation to metabolic health.

The underlying mechanisms behind the association between temperature variability and glucose metabolism are not fully understood, necessitating further exploration. Long-term exposure to higher temperature variability, characterized by unstable weather conditions and frequent temperature fluctuations, may put pressure on the thermoregulatory system, making it more difficult to adjust to the local climate. In response to these environmental temperature changes, the body might redistribute blood flow between cutaneous and visceral vascular beds, potentially influencing glucose levels.^38^ Our findings may indicate that temperature variability potentially contributes to the dysregulation of the crosstalk between the liver and the adipose tissue.^39^ Furthermore, temperature variability may impact fat distribution and activities of fat depots in multiple organs including the brown adipose tissue (BAT). BAT, an insulin-sensitive tissue implicated in thermogenesis, is known to be sensitive to temperature^40^ and might alter its activity under conditions of increased temperature variability. We hypothesize that this would be especially relevant under conditions of global warming that would shift the presence of BAT in populations relative to previous generations. However, it is important to emphasize that these proposed mechanisms remain speculative and have yet to be validated in experimental studies. Future research, particularly mechanistic and experimental work, is needed to clarify the biological pathways linking temperature variability to glucose metabolism.

The present study has several strengths. First, this is the first investigation to explore the effect of temperature variability on glucose metabolism through the use of a longitudinal study design and a large sample size of 4954 observations. The two repeated assessments of the KORA cohort were seven years apart and took place at a time when temperature shifts were already observable.^18^ Second, air temperature was assessed by highly resolved spatiotemporal prediction models^18^ and matched with detailed address information for each participant. Residential address information was initially obtained from official local registration office records and updated for follow-up examinations. This approach minimized misclassification error of residential exposure compared to monitoring station measurements. Third, a wealth of information was collected in the KORA cohort so that we were able to control for potential confounders in the regression models and conduct multiple effect modification analyses.

Our study, however, also had some limitations. First, as this study was confined to a single geographical region, results should be extrapolated to other regions with caution. Also, we found indication that the effects may be potentially stronger in individuals aged 65 years and older, but the present study did not have the statistical power to investigate individuals with underlying cardiometabolic disease or specific treatment regimens separately. Second, using area-level exposure in lieu of individual exposure, misclassification of exposure may have been introduced. Our exposure assessment is further limited by the inability to capture time participants spent away from home (e.g., at work), which is a common challenge in environmental epidemiology studies. We conducted a sensitivity analysis restricted to unemployed participants, the results were consistent with our main findings. Moreover, although temperature data were matched to geocoded participant addresses at the time of clinical assessment and addresses were updated as needed, continuous address histories were not available, raising the possibility that some participants may have changed residence during the study period. However, sensitivity analyses excluding participants who moved during the study period yielded results consistent with the main findings, supporting the robustness of our conclusions. Additionally, the majority of study participants (>73%) resided at their reported addresses for multiple years, further supporting the stability of residential exposure assignment in this study. Third, given the observational nature of this study, the potential for residual and unmeasured confounding cannot be entirely eliminated, thus precluding the possibility of drawing definitive causal inferences. Additionally, the absence of continuous blood glucose monitoring precluded a more comprehensive overview of glucose levels. Future research should consider examining other climatic zones over more extended periods and include populations that might be more vulnerable. Finally, our study lacked detailed dietary data, which prevented adjustment for dietary factors known to influence glucose metabolism. To our knowledge, no published studies have assessed the relationship between annual temperature variability and dietary patterns. Therefore, we cannot exclude the possibility of unmeasured dietary confounding. If dietary habits were to change in response to annual temperature variability, for example, through higher caloric intake or altered food choices during years with unusual temperature swings, such changes could bias our observed associations, most likely in the direction of overestimation if these dietary shifts increase metabolic risk. However, we consider this as rather unlikely in the study region.

In conclusion, our study provides novel evidence that long-term exposure to higher temperature variability is associated with insulin resistance in the general population. The findings of this study may suggest that higher temperature variability will also contribute to increased incidence and severity of type 2 diabetes globally and highlight the detrimental role of climate change for cardiometabolic diseases.

## Supplementary Material



## Notes

(Text S1) Assessment of covariates; (Table S1) measurement of biomarkers of glucose metabolism; (Table S2) nonlinear tests with likelihood ratio tests; (Table S3) annual average temperature and annual temperature variability in the Augsburg region by calendar year; (Table S4) association between temperature variability and HbA1c: segmented regression results with a threshold at 7.5 °C; (Figure S1) exposure–response function of temperature variability and HbA1c; (Figure S2) time trend in glucose metabolism biomarkers in KORA F4; (Figure S3) time trend in glucose metabolism biomarkers in KORA FF4; (Figure S4) Spearman correlations between glucose metabolism biomarkers in KORA F4 and FF4; (Figure S5) Spearman correlations between temperature variability, annual average temperature, and air pollutants in KORA F4 and FF4; (Figure S6) estimation of percent changes in geometric means of glucose metabolism biomarkers with a 1 °C increase in temperature variability modified by smoking status, physical activity, and overweight; (Figure S7) sensitivity analyses: estimation of percent changes in geometric means of glucose metabolism biomarkers with a 1 °C increase in temperature variability; (Figure S8) odd ratios for abnormal glucose metabolism biomarkers per 1 °C increase in temperature variability (PDF)
