# Supplementary material for: Associations of Long-Term Exposure to Temperature Variability with Glucose Metabolism: Results from KORA F4 and FF4
Source: Environ Sci Technol. 2025 Nov 7;59(45):24246–56. doi: 10.1021/acs.est.5c04956 (PMC12631984; doi:10.1021/acs.est.5c04956)
Supplement: Supplementary file 1 [file es5c04956_si_001.pdf]

Supplement

Associations of long-term exposure to temperature variability with glucose metabolism: Results from KORA F4 and FF4

Authors: Wenli Ni[1,2*], Siqi Zhang[1], Christian Herder[3,4,5], Susanne Breitner-Busch[1,2], Kathrin Wolf[1], Minqi Liao[1,2], Nikolaos Nikolaou[1,2], Regina Pickford[1], Wolfgang Koenig[6,7,8], Wolfgang Rathmann[5,9], Lars Schwettmann[10,11], Michael Roden[3,4,5], Barbara Thorand[1,2,5], Annette Peters[1,2,5,7&], Alexandra Schneider[1&]

1. Institute of Epidemiology, Helmholtz Zentrum München - German Research Center for Environmental Health, Neuherberg, D-85764, Germany
2. Institute for Medical Information Processing, Biometry, and Epidemiology, Pettenkofer School of Public Health, LMU Munich, Munich, 81377, Germany
3. Institute for Clinical Diabetology, German Diabetes Center, Leibniz Center for Diabetes Research at Heinrich Heine University Düsseldorf, Düsseldorf, 40225, Germany
4. Division of Endocrinology and Diabetology, Medical Faculty and University Hospital Düsseldorf, Heinrich Heine University Düsseldorf, Düsseldorf, 40204, Germany
5. German Center for Diabetes Research (DZD e.V.), München-Neuherberg, Munich, D-85764, Germany
6. Technical University of Munich, School of Medicine and Health, German Heart Centre, TUM University Hospital, Munich, 80636, Germany
7. Munich Heart Alliance, German Centre for Cardiovascular Research (DZHK e.V.), Partner Site Munich, Munich, 80802, Germany
8. Institute of Epidemiology and Medical Biometry, University of Ulm, Ulm, 89081, Germany

9. Institute for Biometrics and Epidemiology, German Diabetes Centre, Leibniz Centre for Diabetes Research at Heinrich Heine University Düsseldorf, Düsseldorf, 40225, Germany
10. Institute of Health Economics and Health Care Management, Helmholtz Zentrum München - German Research Centre for Environmental Health, Neuherberg, 85764, Germany
11. Department of Health Services Research, School of Medicine and Health Sciences, Carl von Ossietzky Universität Oldenburg, Oldenburg, 26129, Germany

[&]These authors made equal contributions and share last authorship.

Corresponding author:

Wenli Ni

Institute of Epidemiology, Helmholtz Zentrum München - German Research Center for Environmental Health, Neuherberg, D-85764, Germany

Institute for Medical Information Processing, Biometry, and Epidemiology, Pettenkofer School of Public Health, LMU Munich, Munich, 81377, Germany

E-Mail: wni2@bidmc.harvard.edu

Table of Contents:

Text S1. Assessment of covariates

Table S1. Measurement of biomarkers of glucose metabolism

Table S2: Nonlinear tests with likelihood ratio test

Table S3. Annual average temperature and annual temperature variability in Augsburg region by calendar year

Table S4. Association between temperature variability and HbA1c: segmented regression results with a threshold at 7.5°C

Figure S1. Exposure-response function of temperature variability and HbA1c

Figure S2. Time trend in glucose metabolism biomarker in KORA F4

Figure S3. Time trend in glucose metabolism biomarker in KORA FF4

Figure S4. Spearman correlations between glucose metabolism biomarkers in KORA F4 and FF4

Figure S5. Spearman correlations between temperature variability, annual average temperature and air pollutants in KORA F4 and FF4

Figure S6. Estimation of percent changes in geometric mean of glucose metabolism biomarkers with 1°C increase in temperature variability modified by smoking status, physical activity, and overweight.

Figure S7. Sensitivity analyses: Estimation of percent changes in geometric mean of glucose metabolism biomarkers with 1°C increase in temperature variability

Figure S8. Odds ratios for abnormal glucose metabolism biomarkers per 1°C Increase in temperature variability

Summary:

Number of pages: 19

Number of figures: 8

Number of tables: 4

Text S1. Assessment of covariates

Individuals participated in computer-assisted personal interviews and were given questionnaires to assess their demographic/socioeconomic status and lifestyle (age, sex, education, physical activity, alcohol consumption, occupational status, and smoking status); history of disease (diabetes); and medication use (glucose-lowering medication). During the physical examination, anthropometric measurements (height, body weight, waist circumference, and hip circumference) were taken. Body mass index (BMI) was calculated by dividing weight in kilograms by the square of height in meters, and waist-hip ratio was computed by dividing waist circumference in centimeters by hip circumference in centimeters.

Alcohol consumption (g/day) was recorded by self-reported number of alcoholic drinks (beer, wine, or spirits) consumed on the weekday and weekend previous to the examination day. Through the self-reporting of leisure time spent on physical activity throughout both summer and winter, physical activity levels in current analysis were categorized as low physical activity (practically none), medium physical activity (about one hour per week, regular or irregularly), or high physical activity (over two hours per week regularly). Occupational status was determined as employed, self-employed, or in training, and unemployed/retired for those who were unemployed, homemakers, or retired. Smoking was categorized as never smoking, former smoking, and current smoking (including both regular and occasional smoking).

A standard OGTT was administered to those without a previous diagnosis of diabetes. Participants were classified based on glucose tolerance status in accordance with the diagnostic criteria established by the American Diabetes Association[1]. Normal glucose tolerance was defined as fasting glucose <100 mg/dL or OGTT 2h glucose <140 mg/dL. Prediabetes was defined

as isolated impaired fasting glucose (fasting glucose of 100-125 mg/dL), impaired glucose tolerance (OGTT 2h glucose of 140-199 mg/dL), or a combination of both impaired fasting glucose and impaired glucose tolerance. Diabetes was defined as self-reported physician-diagnosed diabetes, the use of glucose-lowering medication, a fasting glucose ≥ 126 mg/dL or OGTT 2h glucose ≥ 200 mg/dL. Serum total cholesterol and high-density lipoprotein were measured using the CHOL Flex and AHDL Flex (Dade Behring, Germany) in KORA F4, respectively; and using the using GLU, LDLC, HDLC, and TRIG Flex assays on a Dimension Vista 1500 instrument (Siemens Healthcare Diagnostics Inc., Newark, USA) or CHOL2 and HDLC3 on Cobas c701/702 instruments (Roche Diagnostics GmbH, Mannheim, Germany) in KORA FF4, respectively[2]. High-sensitivity C-reactive protein (hsCRP) was measured with nephelometric assay on a BN II analyzer (BN II Analyzer, Dade Behring).

Table S1. Measurement of biomarkers of glucose metabolism

Measurements	KORA F4	KORA FF4
Serum Fasting Insulin	Microparticle enzyme immunoassay by electrochemiluminescence immunoassay on Cobas e602 (Roche Diagnostics GmbH, Mannheim, Germany)	Solid-phase enzyme-labeled chemiluminescent immunometric assay on Immulite 2000 (Siemens, Erlangen, Germany) or electrochemiluminescence immunoassay on Cobas e602 (Roche Diagnostics GmbH, Mannheim, Germany)
Serum Fasting Glucose	Hexokinase method on Dimension RxL (GLU Flex, Dade Behring, Deerfield, IL, USA)	Enzymatic, colorimetric method using GLU assay on Dimension Vista 1500 (Siemens) or GLUC3 assay on Cobas c702 (Roche)
HbA1c	Cation-exchange high-performance liquid chromatographic, photometric assays on Adams HA-8160 haemoglobin analysis system (Menarini Diagnostics, Florence, Italy)	Cation-exchange high-performance liquid chromatographic, photometric assays on VARIANT II TURBO Hemoglobin testing system (Bio-Rad Laboratories, Hercules, California, USA)
HOMA-IR	Computed as fasting insulin (μIU/mL) × fasting glucose (mmol/L) / 22.5	Computed as fasting insulin (μIU/mL) × fasting glucose (mmol/L) / 22.5

HOMA-B	Computed as 20 × fasting insulin (µIU/mL) / (fasting glucose [mmol/L] - 3.5)	Computed as 20 × fasting insulin (µIU/mL) / (fasting glucose [mmol/L] - 3.5)
QUICKI	Computed as 1 / (log10 [fasting insulin (µU/mL)] + log10 [fasting glucose (mg/dL)])	Computed as 1 / (log10 [fasting insulin (µU/mL)] + log10 [fasting glucose (mg/dL)])

Table S2. Nonlinear tests with likelihood ratio test.

Biomarkers	*P*-value (LR-test)
Fasting glucose	0.785
2h glucose	0.416
Fasting insulin	0.415
HOMA-IR	0.422
HOMA-B	0.414
QUICKI	0.414
HbA1c	<0.001

LR-test: likelihood ratio test.

Table S3. Annual average temperature and annual temperature variability in Augsburg region by calendar year

Year	Annual average temperature (°C)	Annual temperature variability (°C)
2006	8.70	8.05
2007	9.06	6.56
2008	8.76	6.81
2013	8.17	7.66
2014	9.59	5.98

Table S4. Association between temperature variability and HbA1c: segmented regression results with a threshold at 7.5°C

	%change (95% CI)
Temperature variability < 7.5°C	-0.76 (-1.61-0.10)
Temperature variability ≥ 7.5°C	6.71 (5.78-7.65)

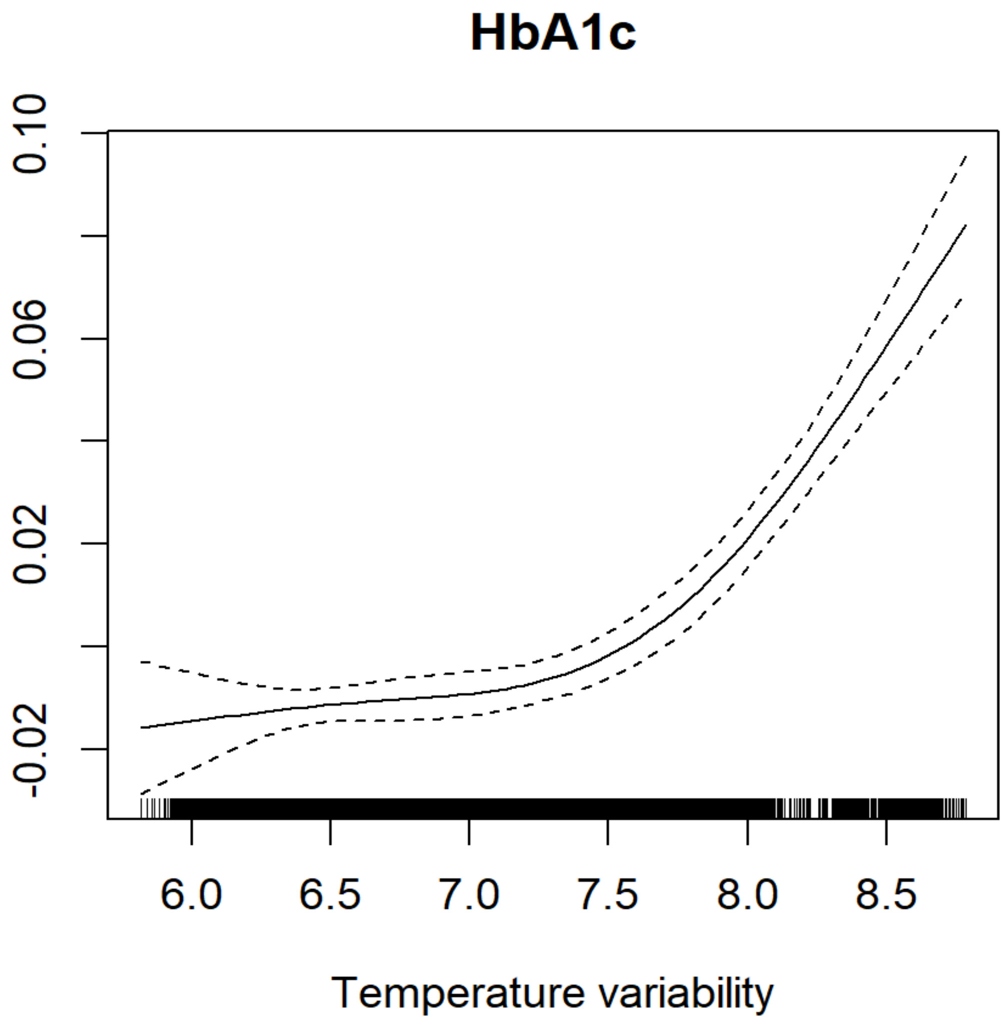

Figure S1. Exposure-response function of temperature variability and HbA1c

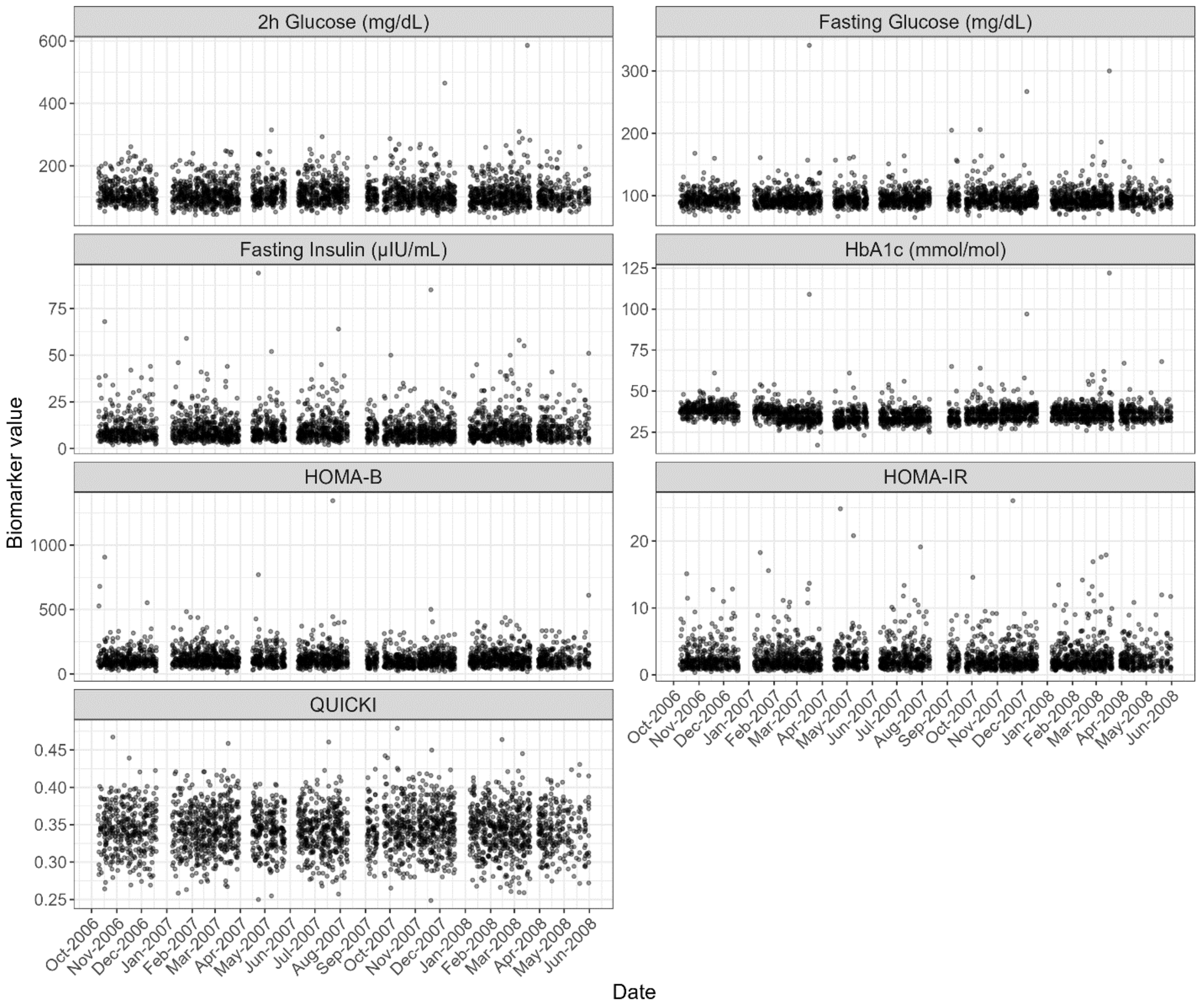

Figure S2. Time trend in glucose metabolism biomarker in KORA F4

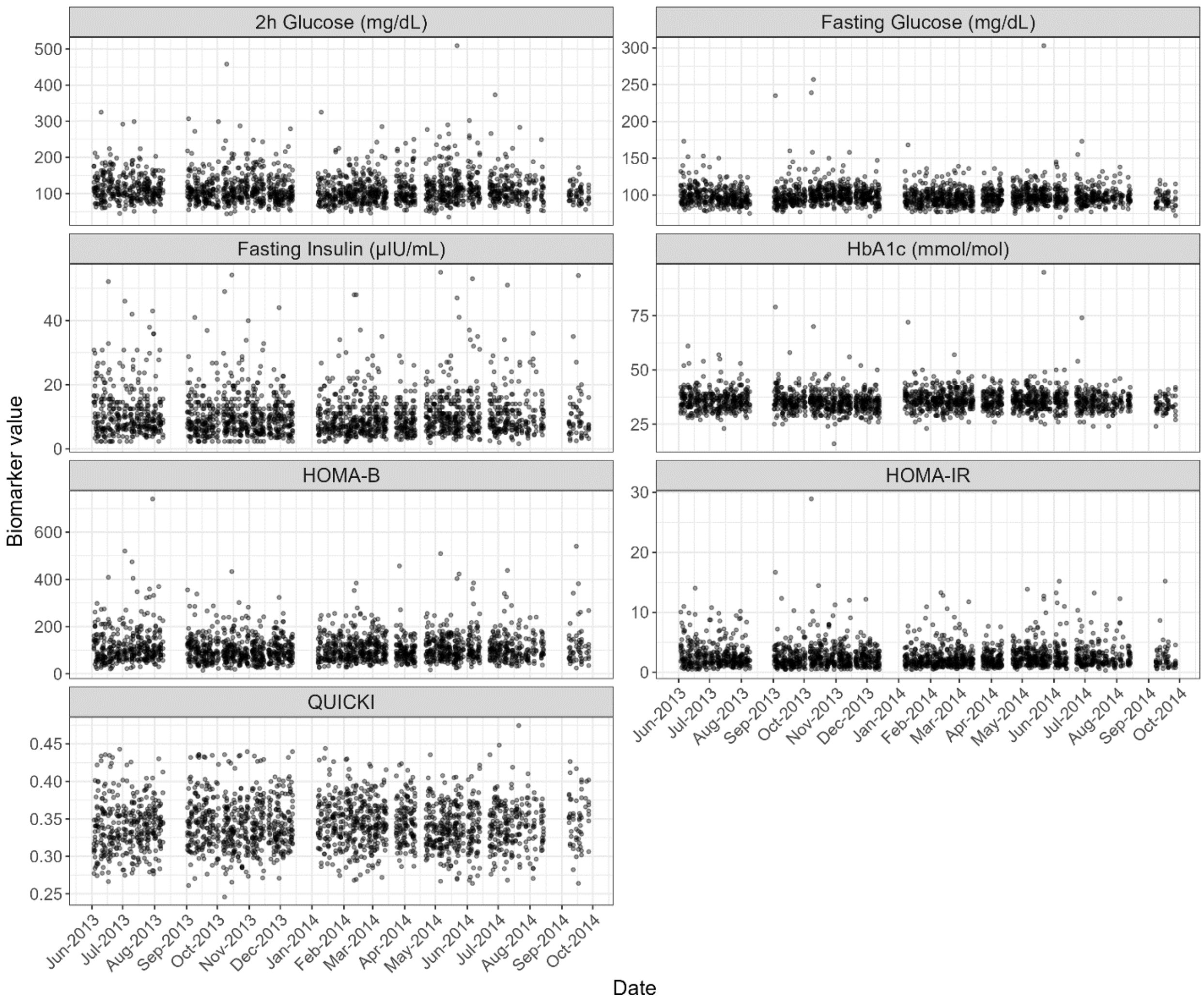

Figure S3. Time trend in glucose metabolism biomarker in KORA FF4

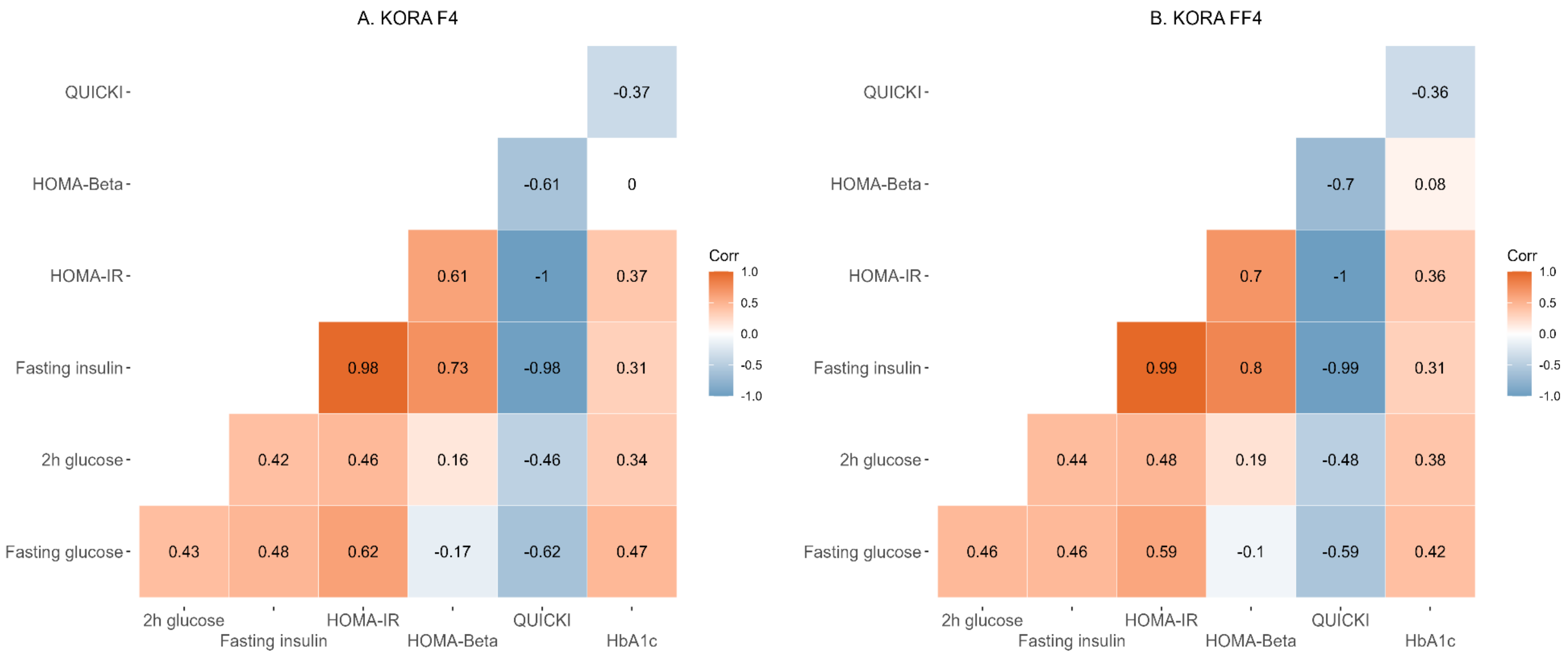

Figure S4. Spearman correlations between glucose metabolism biomarkers in KORA F4 and FF4

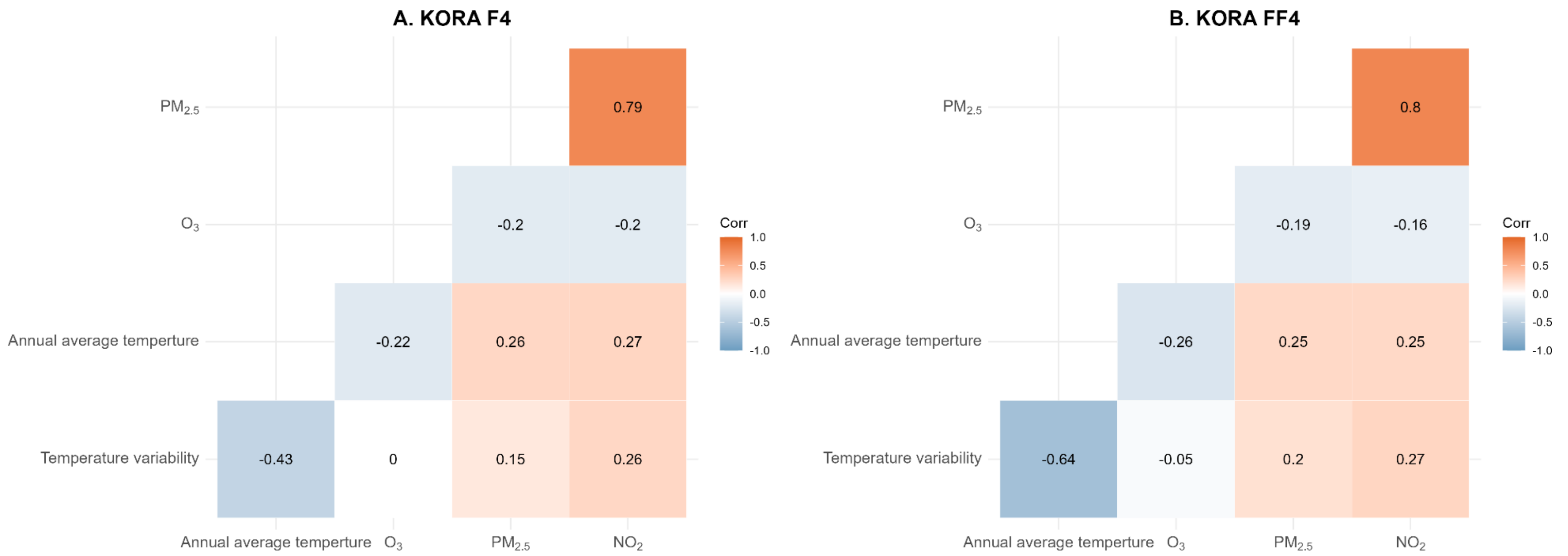

Figure S5. Spearman correlations between temperature variability, annual average temperature and air pollutants in KORA F4 and FF4

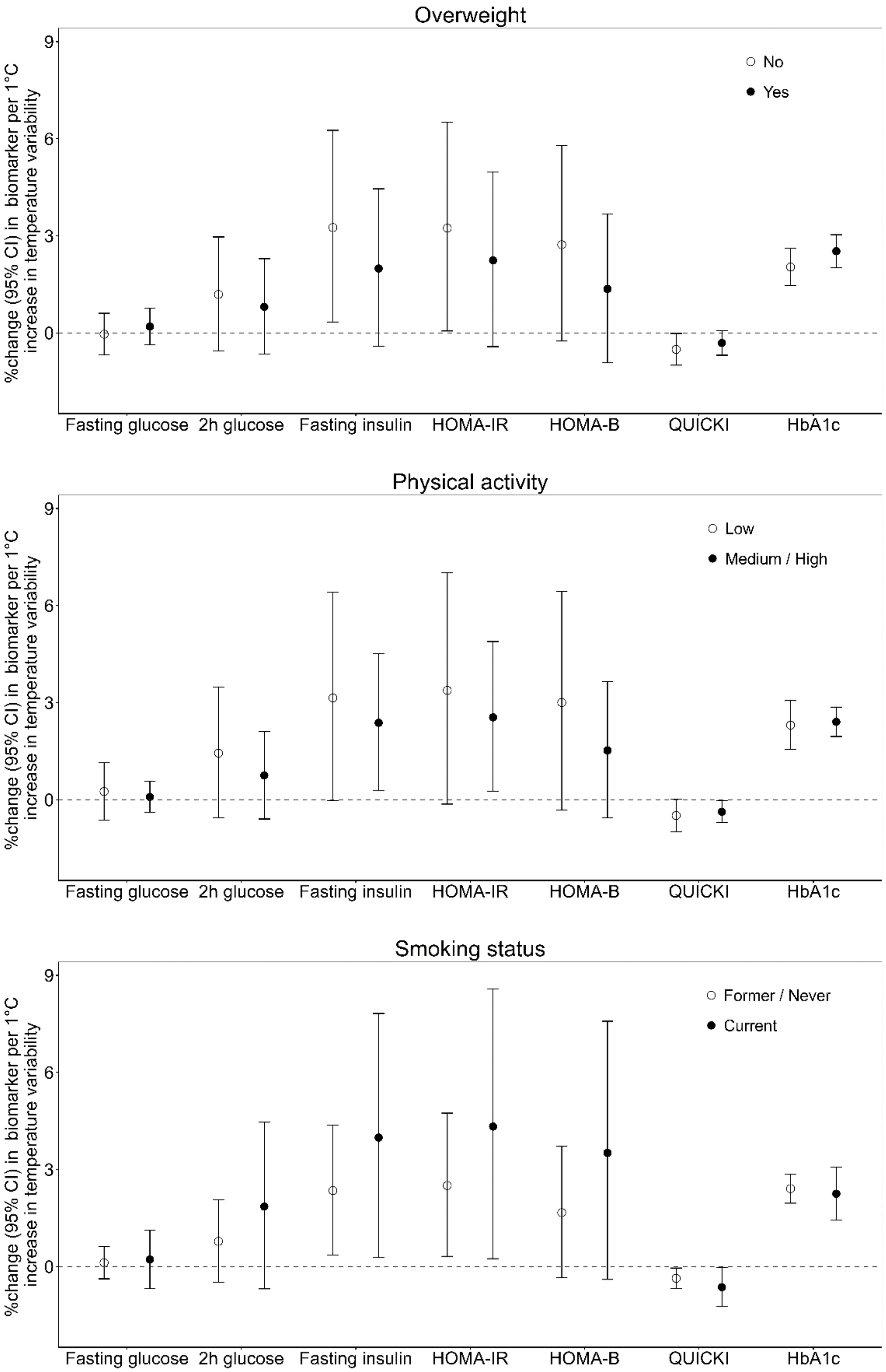

Figure S6. Estimation of percent changes in geometric mean of glucose metabolism biomarkers with 1°C increase in temperature variability modified by smoking status, physical activity, and overweight.

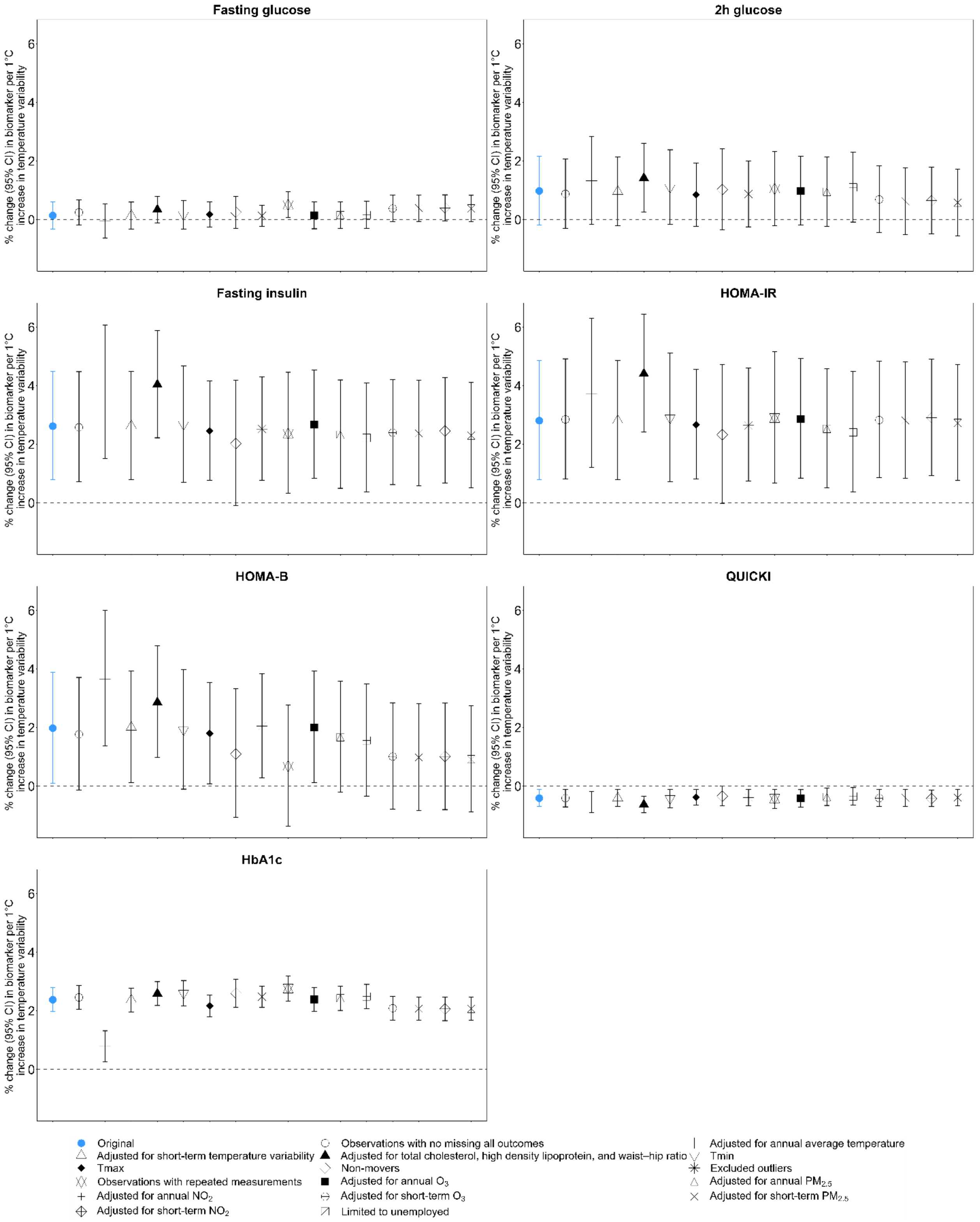

Figure S7. Sensitivity analyses: Estimation of percent changes in geometric mean of glucose metabolism biomarkers with 1°C increase in temperature variability

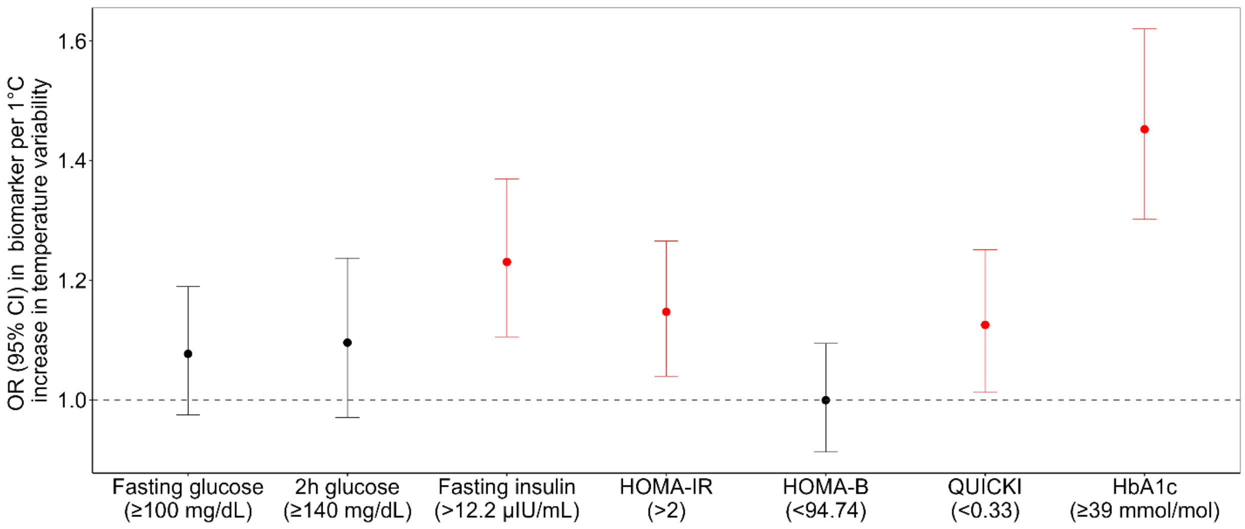

Figure S8. Odds ratios for abnormal glucose metabolism biomarkers per 1°C Increase in temperature variability

Note: "Abnormal" was defined by ADA or literature-based thresholds: fasting glucose (≥100 mg/dL), 2h glucose (≥140 mg/dL), fasting insulin (>12.2 μIU/mL), HOMA-IR (>2), HOMA-B (<94.74), QUICKI (<0.33), and HbA1c (≥39 mmol/mol).

References

1. ElSayed, N. A., Aleppo, G., Aroda, V. R., Bannuru, R. R., Brown, F. M., Bruemmer, D., Collins, B. S., Hilliard, M. E., Isaacs, D., Johnson, E. L., Kahan, S., Khunti, K., Leon, J., Lyons, S. K., Perry, M. L., Prahalad, P., Pratley, R. E., Seley, J. J., Stanton, R. C., Gabbay, R. A., on behalf of the American Diabetes, A. 2. Classification and Diagnosis of Diabetes: Standards of Care in Diabetes-2023. *Diabetes Care* **2023**, *46* (Suppl 1), S19-s40. DOI: 10.2337/dc23-S002

2. Kowall, B., Rathmann, W., Stang, A., Bongaerts, B., Kuss, O., Herder, C., Roden, M., Quante, A., Holle, R., Huth, C., Peters, A., Meisinger, C. Perceived risk of diabetes seriously underestimates actual diabetes risk: The KORA FF4 study. *PloS one* **2017**, *12* (1), e0171152. DOI: 10.1371/journal.pone.0171152
